# Specifying floodwater characteristics in understanding human-mobility response: a comment to Tang *et al.*

**DOI:** 10.1093/nsr/nwad152

**Published:** 2023-05-24

**Authors:** Johan Woltjer

**Affiliations:** Faculty of Spatial Sciences, University of Groningen, Netherlands

One of the most urgent types of disaster risk for cities is rainfall flooding—a risk triggered by climate change and urbanization. Urban actors worldwide are working on a variety of strategies and actions to create flood resilience in cities. With the reality of flood-risk incidents on the increase globally, studies such as Ref. [1] are critical. More mobility research is needed to strengthen our understanding of flood responses, particularly in larger cities. Indeed, we do not currently have sufficient knowledge about actual mobility resilience through the phases of disasters incidents (pre-, during and post-flood). The paper by Tang *et al.* [1] is methodologically innovative in their focus, data use (mobile phone data) and mapping exercise. Studies such as these provide highly useful analysis and legitimate policy suggestions for human mobility in their response to the realities of an extreme urban flood.

However, this kind of work will need to explore further ways to provide clarity on the actual appearance of floods in an urbanized environment. Clarity is crucial for understanding the 2021 Zhengzhou disaster and any other recent and future flood disasters, including for example Bangkok [2]. Assumptions tend to lean strongly on a set of assumed general characteristics of an urban flood. While our understanding of urban population and their capacities and mobilities is relatively strong (e.g. Ref. [3]), detailed insight into actual floodwater behavior in cities will need strong further research.

A more realistic assessment of the level to which for example a pluvial flood impedes mobility requires attention on the following characteristics: floodwater depth (the level to which floodwaters exceed land levels, e.g. in meters), the emerging speed of floodwaters over an urban area (meters per time unit), the timing of water arrival (minutes or hours after a breach or storm), the duration of high water presence (e.g. in hours, days) and exposure (values and types of urban environments and infrastructures affected) (see e.g. Ref. [4]). Also detailed coping behaviors and socio-spatial inequalities related to mobility play a crucial role [5].

The Zhengzhou case [1] suggests that on a generic level floodwaters do not necessarily reduce human mobility fundamentally over an urban region. Evidently, flood-reduced mobility in some areas will be much more severe than elsewhere. It would be helpful to be clearer about when and where certain flood characteristics are seen to reduce mobility more or less strongly. It will be useful, therefore, for further research to evaluate flood disasters by time, magnitude and geography in much more detail. Our understanding and policy decisions would then acknowledge any variation in both flood types and characteristics of urban spaces much more convincingly.

## References

[1] Tang J , Zhao P, Gong Z. Natl Sci Rev 2023; 10: nwad097. 10.1093/nsr/nwad097 PMC10306362 37389148

[2] Laeni N , van den Brink M, Arts J. Cities 2019; 90: 157–67. 10.1016/j.cities.2019.02.002

[3] Qi W , Taylor JE. PLoS One 2016; 11: 0147299. 10.1371/journal.pone.0147299 PMC4731215 26820404

[4] Jonkman B , Jongejan R, Maaskant B. Risk Anal 2011; 31: 282–300. 10.1111/j.1539-6924.2010.01502.x 20883529

[5] Forrest S , Trell E-M, Woltjer J. Trans Inst Br Geogr 2019; 44: 422–36. 10.1111/tran.12279

